# Clinical Study on the Association Between Lipoprotein(a) Levels and Prognosis in Patients With Acute Ischemic Stroke

**DOI:** 10.1002/brb3.71475

**Published:** 2026-05-11

**Authors:** Xinyi Chen, Yueyu Zhang, Yi Tang, Jie Hu, Xun He, Juncang Wu

**Affiliations:** ^1^ Department of Neurology Hefei Hospital Affiliated to Anhui Medical University (Hefei Second People's Hospital) Hefei China; ^2^ The Fifth Clinical Medical School Anhui Medical University Hefei China; ^3^ Department of Neurology Hefei Second People's Hospital Affiliated to Bengbu Medical University Hefei China

## Abstract

**Background:**

Lipoprotein(a) (Lp(a)) is a known cardiovascular risk factor, but its role in acute ischemic stroke (AIS) prognosis remains unclear. This study investigated the association between Lp(a) levels and 3‐month functional outcomes in AIS patients.

**Methods:**

We conducted a retrospective cohort study of 175 AIS patients. Prognosis was assessed using the 3‐month modified Rankin Scale (mRS > 2 defined as poor). Logistic regression and threshold effect models analyzed the association, while ROC curves compared the predictive performance of Lp(a) and NIHSS scores.

**Results:**

Elevated Lp(a) was an independent risk factor for poor 3‐month prognosis (OR = 1.09, *p* = 0.004). A significant threshold effect was identified at 31.2 mg/dL, above which the risk of poor outcomes increased by 79% (*p* = 0.0398). Combining Lp(a) with admission NIHSS scores yielded a significantly higher AUC (0.6820) and specificity (0.9208) than either indicator alone. Sensitivity analyses confirmed the robustness of these findings.

**Conclusion:**

Lp(a) is a stable and independent predictor of poor 3‐month outcomes in AIS patients, exhibiting a clear threshold effect at 31.2 mg/dL. The combined Lp(a)‐NIHSS model enhances prognostic accuracy, supporting Lp(a) as a valuable biomarker for early risk stratification.

## Graphical abstract

Elevated lipoprotein(a) is independently associated with poor 3‐month outcomes in acute ischemic stroke. Combining Lp(a) with NIHSS improves prognostic accuracy.

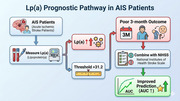

## 1 Introduction

Acute ischemic stroke (AIS) is one of the leading neurological causes of disability and death worldwide (Hong et al. 2025; Nam et al. 2024; J. Wang et al. 2022). In clinical research, accurately distinguishing different ischemic stroke subtypes within the acute stroke population (e.g., cardioembolic stroke, lacunar infarction, cryptogenic infarction, primary cerebral infarction, atherosclerotic thrombotic infarction) holds significant importance (Gasull and Arboix 2022). Similarly, patients often experience varying degrees of neurological deficits after onset, such as limited limb movement and impaired language function. These impairments not only severely diminish patients' quality of life but also impose substantial medical burdens and economic pressures on families and society.

Lipoprotein(a) (Lp(a)), as a unique lipoprotein molecule, possesses a distinctive structure composed of low‐density lipoprotein (LDL)‐like particles linked to apolipoprotein(a) (Apo(a)) via disulfide bonds. The kringle IV domain of Apo(a) exhibits high homology with plasminogen. This structural feature confers dual biological effects: promoting atherosclerosis while inhibiting fibrinolysis (Kosmas et al. 2024; Panagiotopoulos et al. 2025; Pavlatos and Kalra 2024). Previous epidemiological studies have confirmed that elevated Lp(a) levels constitute an independent risk factor for cardiovascular diseases such as coronary heart disease and aortic valve stenosis (Arsenault and Kamstrup 2022; Bhatia and Wilkinson 2022; Raitakari et al. 2022), and its levels are primarily regulated by genetic factors, showing little significant influence from lifestyle interventions such as diet or exercise. This characteristic lends it a stable reference value in disease risk prediction. In recent years, increasing research has focused on the potential association between Lp(a) and acute cerebral infarction prognosis, suggesting it may participate in the pathological processes driving disease progression during the acute phase of cerebral infarction (Hoshino et al. 2025; Xu et al. 2022).

Based on the current research landscape and clinical needs, this study aims to systematically investigate the association between Lp(a) levels and patient prognosis through retrospective analysis of clinical data from acute cerebral infarction patients. The study will employ univariate and multivariable logistic regression to adjust for confounding factors, determining whether Lp(a) is an independent risk factor for poor prognosis. Threshold effect analysis will identify the critical Lp(a) level influencing prognosis, while receiver operating characteristic (ROC) curves will compare the predictive efficacy of Lp(a) versus the National Institutes of Health Stroke Scale (NIHSS) scores. These findings are expected to provide a novel biomarker for prognosis assessment in acute cerebral infarction patients, offering a scientific basis for developing precise risk stratification strategies and intervention plans in clinical practice, ultimately contributing to improved long‐term quality of life for patients.

## 2 Methods

### 2.1 Study Design and Participants

This was a retrospective cohort study that enrolled patients with AIS admitted to the Department of Neurology of our hospital between January 2024 and June 2025. Inclusion criteria required all of the following: age 18–85 years; diagnosis of ischemic stroke meeting the World Health Organization criteria, with confirmed ischemic lesions (including non‐cardiogenic embolic stroke) on CT or MRI; admission within 24 h of symptom onset with varying degrees of neurological deficits (e.g., limb movement disorder, decreased muscle strength, unsteady gait); normal cognitive function, clear consciousness, stable vital signs, and ability to cooperate with treatment and study assessments. Exclusion criteria were any of the following: stroke caused by non‐atherosclerotic intracranial stenosis (e.g., arterial dissection, moyamoya disease, vasculitis, radiation‐induced vasculopathy); ipsilateral intracranial and/or extracranial angioplasty within 1 month before enrollment or planned within 1 month after enrollment; history of thrombectomy; intracranial hemorrhage (including hemorrhagic transformation) within 3 months before enrollment; intracranial tumor, cerebral aneurysm, or cerebral arteriovenous malformation; severe liver dysfunction or renal failure; missing clinical data, laboratory data, or poor‐quality neuroimaging. According to these criteria, 23 patients were excluded (detailed in Table S2), including 12 who received thrombolysis or thrombectomy (6.1%), 3 who were lost to follow‐up or died (1.5%), 6 with missing Lp(a) data (3.0%), and 2 with missing complication information (1.0%). The overall exclusion rate was 11.6%, leaving 175 patients for final analysis. All patients or their legal representatives provided written informed consent. This study was approved by the Medical Ethics Committee of Hefei Second People's Hospital, Anhui Medical University Affiliated Hefei Hospital (Approval No. 2023‐Research‐006‐01).

### 2.2 Clinical Data Collection

Trained neurologists conducted face‐to‐face interviews to collect the following data: demographic information (including age and gender), stroke risk factors (including smoking or alcohol consumption, physical activity, hypertension, diabetes, prior stroke, etc.), and pre‐ and post‐treatment modified Rankin Scale (mRS) and NIHSS scores. At admission, laboratory tests (including triglycerides [TG], LDL cholesterol [LDL‐C], C‐reactive protein, D‐dimer, and Lp(a)) were performed: Fasting venous blood was collected from patients, serum was separated by centrifugation, and immunoturbidimetry (precise and efficient) was used for measurement, with a few using enzyme‐linked immunosorbent assay (ELISA). Serum Lp(a) concentrations were quantified using fully automated biochemical analyzers. Participants avoided high‐fat meals and strenuous exercise prior to testing. Carotid Doppler ultrasound procedure: The examinee lies supine with the head turned to expose the neck. After applying a coupling agent to the ultrasound probe, it is slid along the carotid artery pathways on both sides of the neck. The common carotid artery, internal carotid artery, and external carotid artery are sequentially scanned to measure intima‐media thickness (IMT), observe vascular wall structure, detect plaque presence, and assess blood flow. In addition, the use of antiplatelet agents or statins during the patient's hospitalization is documented. The severity of white matter lesions is assessed using the Fazekas scale (3 grades), and stroke subtypes are classified according to the TOAST classification criteria (macroarterial atherosclerotic, microarterial occlusive, other types).

### 2.3 Neurological Assessment and Outcome Definition

All patients underwent neurological deficit assessment using the NIHSS upon admission. The primary outcome measure was neurological status at 3 months post‐onset, evaluated using the mRS. Patients with mRS ≤ 2 were classified as the favorable prognosis group, while those with mRS > 2 constituted the unfavorable prognosis group.

### 2.4 Research Methods

This retrospective cohort analysis included 175 patients with acute cerebral infarction, categorized into a favorable prognosis group (*n* = 101) and an unfavorable prognosis group (*n* = 74) based on clinical outcomes. For data processing, missing values in clinical records, laboratory tests, and imaging data were handled using complete case analysis. Only patients with complete data for Lp(a), prognostic indicators, and key covariates (gender, age, underlying diseases, etc.) were included to ensure statistical reliability. Baseline data were collected for all subjects. Quantitative data are described as “mean ± standard deviation (mean ± SD),” while qualitative data are presented as “number (percentage) (*n* (%)).” Univariate analysis was used to screen potential prognostic factors. *logistic regression analysis was employed to construct unadjusted models (Model 1) and adjusted models (Model 3), controlling for gender, age, smoking, drinking, hypertension, diabetes, heart disease, stroke, weight, height, physical activity, systolic blood pressure (SBP), diastolic blood pressure (DBP), and TOAST classification (Model 3*). The association and trend (*p* for trend) between Lp(a) tertiles (low, medium, high) and prognosis were analyzed concurrently. Threshold effect analysis was used to determine the breakpoint value of Lp(a) influencing prognosis. ROC curve analysis assessed the predictive value of admission NIHSS scores and Lp(a) for prognosis, calculating area under the curve (AUC), 95% confidence interval (95% CI), optimal threshold, specificity, and sensitivity. All statistical analyses were performed using SPSS software and R 4.4.2. A *p*‐value < 0.05 was considered statistically significant.

## 3 Results

### 3.1 Baseline Characteristics of Participants

This study included 175 participants, whose baseline characteristics are summarized in Table 1, divided into a “favorable prognosis” group (*n* = 101) and an “unfavorable prognosis” group (*n* = 74). Significant differences existed between the two groups across multiple indicators (*p* < 0.05). Compared with the favorable prognosis group, The “poor prognosis” group exhibited higher levels of Lp(a) (23.42 ± 8.36 vs. 18.64 ± 6.85), TG (1.73 ± 1.62 vs. 1.30 ± 0.71), LDL (3.03 ± 0.83 vs. 2.61 ± 0.86), D‐dimer, C‐reactive protein, and SBP compared with the “favorable prognosis” group. Neurological function scores were also worse (admission NIHSS: 5.22 ± 4.30 vs. 3.89 ± 3.37; discharge NIHSS: 3.50 ± 3.34 vs. 2.02 ± 1.33). Regarding demographics and medical history, the “poor prognosis” group had older age (73.35 ± 10.93 vs. 63.77 ± 12.54) and a higher proportion of females (51.35% vs. 32.67%); antiplatelet therapy: 157 patients (89.71%) received treatment, with significantly higher usage in the favorable prognosis group (97.03%) compared to the poor prognosis group; statin therapy: 159 patients (90.86%) received treatment, with markedly higher usage in the favorable prognosis group (98.02%) compared to the poor prognosis group (81.08%); ischemic lacunar stroke: 99 patients (56.57%). Both stroke subtypes showed significant association with patient prognosis (*p* < 0.001). Patients in the poor prognosis group had significantly higher prevalence rates of hypertension, diabetes mellitus, and prior stroke history. In contrast, there were no significant differences between the two groups in terms of body weight, height, DBP, physical activity level, history of heart disease, smoking history, or alcohol consumption history (all *p* > 0.05).

**TABLE 1:** Participant characteristics.

Variables	Total	Good prognosis	Poor prognosis	*p*‐value
Triglycerides, mean ± SD	1.49 ± 1.19	1.30 ± 0.71	1.73 ± 1.62	0.019
Low‐density lipoprotein, mean ± SD	2.79 ± 0.87	2.61 ± 0.86	3.03 ± 0.83	0.001
D‐dimer, mean ± SD	0.89 ± 1.81	0.61 ± 1.71	1.28 ± 1.87	0.014
C‐reactive protein, mean ± SD	3.26 ± 4.37	2.59 ± 4.75	4.18 ± 3.64	0.017
Right carotid artery IMT, mean ± SD	0.89 ± 0.19	0.81 ± 0.18	0.99 ± 0.15	< 0.001
Left carotid artery IMT, mean ± SD	0.89 ± 0.19	0.80 ± 0.17	1.01 ± 0.15	< 0.001
Admission mRS, mean ± SD	2.44 ± 1.16	2.16 ± 1.09	2.82 ± 1.14	< 0.001
Admission NIHSS score, mean ± SD	4.45 ± 3.84	3.89 ± 3.37	5.22 ± 4.30	0.024
Out NIHSS score, mean ± SD	2.65 ± 2.50	2.02 ± 1.33	3.50 ± 3.34	< 0.001
Out mRS, mean ± SD	1.95 ± 1.20	1.37 ± 0.88	2.76 ± 1.12	< 0.001
Lipoprotein(a), mean ± SD	20.66 ± 7.87	18.64 ± 6.85	23.42 ± 8.36	< 0.001
Age, mean ± SD	67.82 ± 12.77	63.77 ± 12.54	73.35 ± 10.93	< 0.001
Heavy, mean ± SD	63.98 ± 12.17	64.32 ± 11.76	63.51 ± 12.79	0.668
Height, mean ± SD	1.64 ± 0.08	1.64 ± 0.08	1.64 ± 0.08	0.648
Systolic blood pressure, mean ± SD	134.43 ± 21.07	130.20 ± 17.12	140.20 ± 24.45	0.003
Diastolic blood pressure, mean ± SD	79.65 ± 11.70	79.74 ± 10.61	79.53 ± 13.11	0.905
Gender, *n* (%)				0.013
Female	71 (40.57)	33 (32.67)	38 (51.35)	
Male	104 (59.43)	68 (67.33)	36 (48.65)	
Activity, *n* (%)				0.948
Yes	88 (50.29)	51 (50.50)	37 (50.00)	
No	87 (49.71)	50 (49.50)	37 (50.00)	
Hypertension, *n* (%)				< 0.001
Yes	104 (59.43)	47 (46.53)	57 (77.03)	
No	71 (40.57)	54 (53.47)	17 (22.97)	
Diabetes, *n* (%)				< 0.001
Yes	59 (33.71)	19 (18.81)	40 (54.05)	
No	116 (66.29)	82 (81.19)	34 (45.95)	
Heart disease, *n* (%)				0.417
Yes	18 (10.29)	12 (11.88)	6 (8.11)	
No	157 (89.71)	89 (88.12)	68 (91.89)	
Stroke, *n* (%)				0.005
Yes	94 (53.71)	45 (44.55)	49 (66.22)	
No	81 (46.29)	56 (55.45)	25 (33.78)	
Smoke, *n* (%)				0.122
Yes	33 (18.86)	23 (22.77)	10 (13.51)	
No	142 (81.14)	78 (77.23)	64 (86.49)	
Drink, *n* (%)				0.49
Yes	45 (25.71)	24 (23.76)	21 (28.38)	
No	130 (74.29)	77 (76.24)	53 (71.62)	
TOAST classification, *n* (%)				0.007
Large artery atherosclerosis type	73 (41.71)	32 (31.68)	41 (55.41)	
Small artery occlusion type	74 (42.29)	50 (49.50)	24 (32.43)	
Other types	28 (16.00)	19 (18.81)	9 (12.16)	
Fazekas, *n* (%)				< 0.001
Level 1	79 (45.14)	55 (54.46)	24 (32.43)	
Level 2	45 (25.71)	30 (29.70)	15 (20.27)	
Level 3	51 (29.14)	16 (15.84)	35 (47.30)	
Stroke type, *n* (%)				< 0.001
Non‐lacunar ischemic stroke	76 (43.43)	31 (30.69)	45 (60.81)	
Lacunar ischemic stroke	99 (56.57)	70 (69.31)	29 (39.19)	
Antiplatelet, *n* (%)				< 0.001
No	18 (10.29)	3 (2.97)	15 (20.27)	
Yes	157 (89.71)	98 (97.03)	59 (79.73)	
Statin use, *n* (%)				< 0.001
No	16 (9.14)	2 (1.98)	14 (18.92)	
Yes	159 (90.86)	99 (98.02)	60 (81.08)	

*Note*: Quantitative data (such as TG, LDL, age, etc.) are presented as “mean ± SD”; categorical data (such as gender, history of hypertension, TOAST classification, etc.) are presented as “*n* (%)”.

### 3.2 Univariate Analysis of the Association Between Lp(a) and Prognosis in Acute Cerebral Infarction

Univariate analysis results in Table 2 indicate that Lp(a), as a key prognostic factor in acute cerebral infarction, exhibits an overall mean value of 20.66 ± 7.87. It shows a significant positive correlation with the risk of poor patient prognosis (OR = 1.09, 95% CI: 1.04–1.13, *p* < 0.0001). This indicates that for every 1‐unit increase in Lp(a) level, the risk of poor prognosis increases by 9%. In addition, univariate analysis identified other prognostic factors: age (OR = 1.07, *p* < 0.0001), SBP (OR = 1.02, *p* = 0.0025), and history of prior stroke (OR = 0.41, *p* = 0.0049, compared to those without history) were significant risk factors. Male gender (OR = 0.46, *p* = 0.0136, compared to females), absence of hypertension (OR = 0.26, *p* < 0.0001), and absence of diabetes (OR = 0.20, *p* < 0.0001) were protective factors. DBP and alcohol consumption status showed no significant association with prognosis in acute cerebral infarction patients (*p* > 0.05 for all).

**TABLE 2:** Univariate analysis of lipoprotein(a) and outcome prognosis.

	Statistics	Prognosis
Lp(a)	20.66 ± 7.87	1.09 (1.04, 1.13) 0.0001
SBP	134.43 ± 21.07	1.02 (1.01, 1.04) 0.0025
DBP	79.65 ± 11.70	1.00 (0.97, 1.02) 0.9039
Age	67.82 ± 12.77	1.07 (1.04, 1.10) < 0.0001
Drink		
Yes	45 (25.71%)	Ref.
No	130 (74.29%)	0.79 (0.40, 1.56) 0.4905
Gender		
Female	71 (40.57%)	Ref.
Male	104 (59.43%)	0.46 (0.25, 0.85) 0.0136
Hypertension		
Yes	104 (59.43%)	Ref.
No	71 (40.57%)	0.26 (0.13, 0.51) < 0.0001
Diabetes		
Yes	59 (33.71%)	Ref.
No	116 (66.29%)	0.20 (0.10, 0.39) < 0.0001
Stroke		
Yes	94 (53.71%)	Ref.
No	81 (46.29%)	0.41 (0.22, 0.76) 0.0049

### 3.3 Logistic Regression Results for the Association Between Lp(a) and Prognosis in Acute Cerebral Infarction

As shown in Table 3, the results of the multivariate logistic regression analysis indicate that Lp(a) is significantly positively associated with the risk of poor prognosis in patients with acute cerebral infarction. This association remains stable after adjusting for multiple confounding factors: in Model 1 (without adjusting for any confounding factors), the odds ratio (OR) (95% CI) for the continuous variable of Lp(a) was 1.09 (1.04, 1.13), *p* = 0.0001; after adjusting for confounders in Model 2, the OR (95% CI) for the continuous Lp(a) variable remained 1.09 (1.03, 1.15), *p* = 0.0043; *Model 3, which further adjusted for TOAST classification based on Model 2, showed no substantial change in the association strength or statistical significance of the continuous Lp(a) variable, with an OR (95% CI) of 1.09 (1.03, 1.15), p = 0.0042. In a three‐group analysis of Lp(a) (low group as reference), the high group showed a significantly increased risk of poor prognosis: OR (95% CI) was 3.21 (1.51, 6.86), p = 0.0025 in Model 1; OR (95% CI) in Model 2: 3.89 (1.33, 11.39), p = 0.0130; OR (95% CI) in Model 3 was 3.90 (1.33, 11.45), p = 0.0132. Trend tests yielded p‐values of 0.0022, 0.0121, and 0.0117, respectively, indicating a significant increasing trend in adverse prognosis risk with higher Lp(a) levels*.

**TABLE 3:** Multivariate logistic regression analysis of lipoprotein(a) and outcome prognosis.

Exposure	Model 1	Model 2	Model 3
	OR (95% CI) *p*‐value	OR (95% CI) *p*‐value	OR (95% CI) *p*‐value
Lp(a) continuous variable	1.09 (1.04, 1.13) 0.0001	1.09 (1.03, 1.15) 0.0043	1.09 (1.03, 1.15) 0.0042
Lp(a) grouping variable			
Low group	Ref.	Ref.	Ref.
Medium group	1.00 (0.46, 2.17) 1.0000	1.63 (0.56, 4.76) 0.3682	1.54 (0.51, 4.61) 0.4423
High group	3.21 (1.51, 6.86) 0.0025	3.89 (1.33, 11.39) 0.0130	3.90 (1.33, 11.45) 0.0132
Trend	0.0022	0.0121	0.0117

*Note*: Model 1 adjust for: none; Model 2 adjust for: gender, age, smoke, drink, hypertension, diabetes, heart disease, stroke, weight, height, activity, SBP, and DBP; Model 3 adjust for: gender, age, smoke, drink, hypertension, diabetes, heart disease, stroke, weight, height, activity, SBP, DBP, and TOAST classification.

Abbreviations: CI, confidence interval; OR, odds ratio; Ref., reference.

### 3.4 Nonlinear Model Results for the Association Between Lp(a) and Acute Cerebral Infarction Prognosis

**FIGURE 1:**
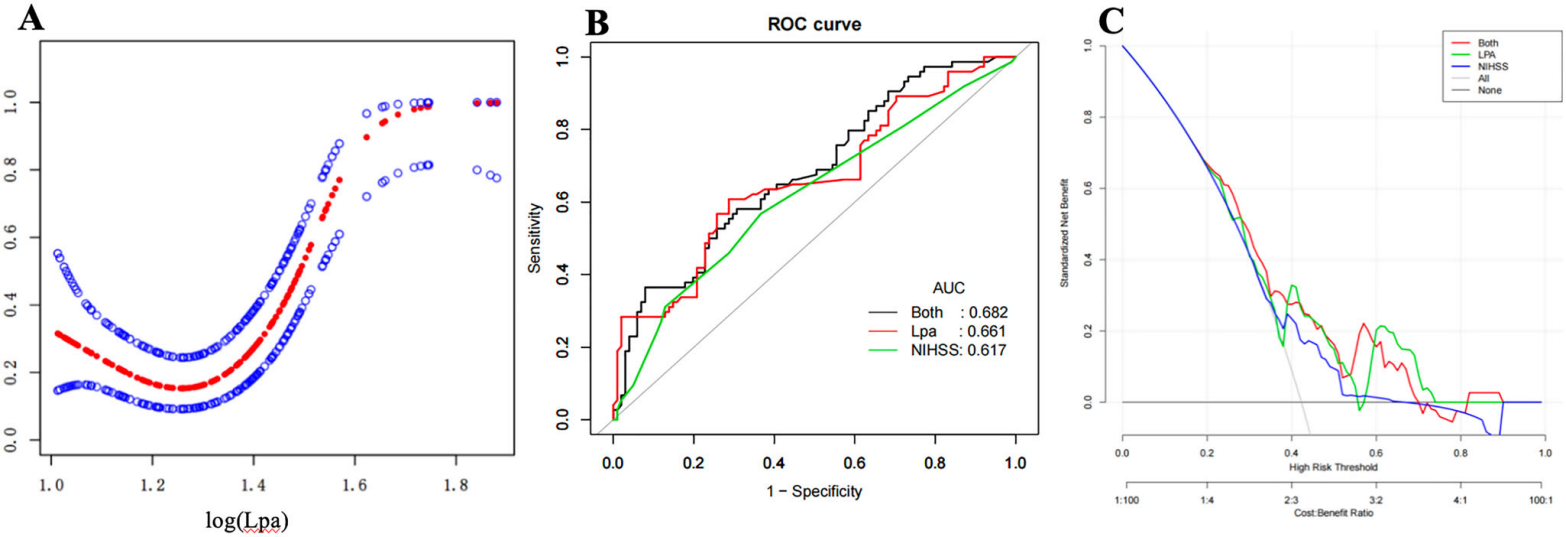
Association between lipoprotein(a) (Lp(a)) levels and 3‐month prognosis in patients with acute ischemic stroke (AIS). (A) Restricted cubic spline curve illustrating the nonlinear relationship between log‐transformed Lp(a) levels and the risk of poor 3‐month functional outcomes, adjusted for relevant confounding factors. Red dots represent the fitted risk estimates, and blue dots indicate the 95% confidence intervals. (B) Receiver operating characteristic (ROC) curves comparing the prognostic predictive performance of Lp(a), admission National Institutes of Health Stroke Scale (NIHSS) score, and the combined Lp(a) + NIHSS model. The combined model demonstrated the highest predictive value (AUC = 0.682). (C) Decision curve analysis (DCA) showing the net clinical benefit of the combined model, Lp(a) alone, and NIHSS alone across different threshold probabilities, indicating superior clinical utility of the combined model.

The threshold effect analysis results in Table 4 indicate that after adjusting for confounding factors including Model 3, a significant inflection point *K* = 31.2 exists in the association between Lp(a) and acute cerebral infarction prognosis. When Lp(a) < 31.2, no significant association with prognosis was observed (effect size 1.05, 95% CI: 0.98–1.13, *p* = 0.1742); when Lp(a) > 31.2, each 1‐unit increase in Lp(a) significantly increased the risk of poor prognosis by 79% (effect size 1.79, 95% CI: 1.03–3.13, *p* = 0.0398), with a log‐likelihood ratio test *p* = 0.045, indicating a stable and reliable threshold effect. Similarly, the curve‐fitting model in Figure 1 (adjusted for relevant confounders) visually demonstrates the nonlinear dose–response relationship between log‐transformed Lp(a) and prognostic risk, further validating the authenticity of the aforementioned threshold effect.

**TABLE 4:** Threshold effect analysis of lipoprotein(a) and outcome prognosis.

Outcome	Prognosis
Breakpoint (*K*)	31.2
< *K* Segment Effect 1	1.05 (0.98, 1.13) 0.1742
> *K* Segment Effect 2	1.79 (1.03, 3.13) 0.0398
Log‐likelihood ratio test	0.045

*Note*: Adjust for: gender, age, smoke, drink, hypertension, diabetes, heart disease, stroke, weight, height, activity, SBP, DBP, and TOAST classification.

### 3.5 ROC Prediction Model for Lp(a) and Acute Cerebral Infarction Prognosis

Results from Table 5, Figure 1, and Table S1 indicate that Lp(a) serves as a prognostic predictor for acute cerebral infarction. Its area under the ROC curve (AUC) was 0.6611 (95% CI: 0.5782–0.7440), with an optimal threshold of 19.88 yielding a specificity of 0.7129 and sensitivity of 0.6081, and accuracy of 0.6686, indicating moderate predictive value of Lp(a) alone for acute cerebral infarction prognosis. The combined predictive model of “Lp(a) + admission NIHSS score” demonstrated superior performance, with an AUC of 0.6820 (95% CI: 0.6026–0.7614), significantly higher than the standalone Lp(a) model and the standalone admission NIHSS score model (AUC = 0.6169, optimal cutoff 3.5, specificity 0.6337, sensitivity 0.5676). Furthermore, the combined model demonstrated significantly higher specificity (0.9208), diagnostic OR (6.6782), and positive predictive value (0.7714) compared to the Lp(a) model alone (diagnostic OR 3.8526, positive predictive value 0.6081), suggesting that combining Lp(a) with admission NIHSS score enables more accurate prediction of acute cerebral infarction prognosis, with significantly stronger predictive capability than either single indicator alone.

**TABLE 5:** ROC curve analysis of lipoprotein(a) and outcome prognosis.

ROC analysis for continuous predictor						
Test	ROC area (AUC)	95% CI low	95% CI up	Best threshold	Specificity	Sensitivity
Both	0.6820	0.6026	0.7614	0.2759	0.9208	0.3649
LPA	0.6611	0.5782	0.7440	19.8800	0.7129	0.6081
Admission NIHSS score	0.6169	0.5331	0.7007	3.5000	0.6337	0.5676

### 3.6 Sensitivity Analysis

In this study, multiple sensitivity analyses were performed on the 175 enrolled patients with AIS to verify the robustness of the association between Lp(a) and poor prognosis at 3 months. Table S5 presents the patient exclusion process and missing data details. A total of 23 patients were excluded, including 12 who received thrombolysis or thrombectomy (6.1%), 3 who were lost to follow‐up or died (1.5%), 6 with missing Lp(a) data (3.0%), and 2 with missing complication information (1.0%). The overall exclusion rate was 11.6%, leaving 175 patients with a high degree of data completeness. As shown in Table S3 and Figure S1 (forest plot), after including patients who received thrombolysis or thrombectomy and additionally controlling for these treatments based on Model 3 of logistic regression, the high Lp(a) group remained significantly associated with an increased risk of poor prognosis (OR = 3.44, 95% CI: 1.21–9.73, *p* = 0.0202), with a significant dose–response trend (*p* for trend = 0.0155).

Table S4 and Figure S2 (forest plot) demonstrate that after including patients with missing Lp(a) data, the results remained consistent. In Model 3, the high Lp(a) group still had a significantly higher risk of poor prognosis (OR = 4.14, 95% CI: 1.40–12.25, *p* = 0.0102), and the trend test was also statistically significant (*p* for trend = 0.0090). Furthermore, the *E*‐value sensitivity analysis showed that the point estimate (OR = 3.90) corresponded to an *E*‐value of 7.26, and the lower bound of the 95% CI (1.33) corresponded to an *E‐*value of 1.99. This suggests that unmeasured confounding would need to be strongly associated with both Lp(a) and poor prognosis to explain away the observed association. Collectively, these sensitivity analyses confirm that the association between high Lp(a) levels and 3‐month poor prognosis in patients with AIS is stable, reliable, and robust.

## 4 Discussion

In this study, elevated Lp(a) levels were independently associated with an increased risk of unfavorable functional outcomes at 3 months in patients with AIS. This association remained robust after adjustment for multiple confounders, including TOAST classification, and demonstrated a clear dose–response relationship. A notable finding was the identification of a threshold effect at 31.2 mg/dL, above which the risk of poor outcomes increased significantly. This nonlinear relationship suggests that Lp(a) may exert a clinically meaningful effect only beyond a certain level. Furthermore, the combined model incorporating Lp(a) and admission NIHSS score demonstrated superior predictive performance compared with either variable alone, highlighting the added value of integrating biochemical and clinical indicators.

Previous studies have found, for example, that Jiang et al. (2021) in a cohort study in China observed an association between elevated Lp(a) levels and adverse functional outcomes at 3 months and 1 year in patients with ischemic stroke. After adjusting for confounding factors, the highest quartile (Q4) of Lp(a) compared to the lowest quartile (Q1) had an OR of 1.33 for 3‐month adverse outcomes and an OR of 1.25 for 1‐year outcomes. The high Lp(a)/high Lp‐PLA2 group exhibited the highest risk of adverse outcomes at 3 months (OR 1.21) and 1 year (OR 1.25), suggesting a potential synergistic increase in the risk of adverse functional outcomes in ischemic stroke patients (Jiang et al. 2021). A prospective study by Yuan et al. (2024) found that elevated Lp(a) levels remained associated with poor functional outcomes in AIS patients even with well‐controlled LDL‐C (adjusted hazard ratio [aHR] 1.31, 95% CI: 1.09–1.59). Similarly, R. Wang et al. (2024) investigated the association between serum Lp(a) levels and early neurological deterioration (END) after thrombolysis. They found that the proportion of high LP(a) (≥ 300 mg/L) in the END group (38.3%) was significantly higher than in the non‐END group (22.2%) (*p* < 0.005). After adjusting for confounding factors including age, sex, smoking history, and stroke subtype via multivariate logistic regression analysis, elevated LP(a) emerged as an independent risk factor for post‐thrombolysis END (OR = 3.154, 95% CI: 1.067–9.322, *p* = 0.038), suggesting that LP(a) ≥ 300 mg/L may be associated with the occurrence of END after thrombolysis in AIS patients and could serve as a predictive indicator for this adverse outcome(R. Wang et al. 2024).

A prospective study by Hoshino et al. (2025) similarly indicated that the incidence of cardiovascular and cerebrovascular events (19.1%) was higher in the Lp(a) > 15 mg/dL group than in the low Lp(a) group (10.7%). Lp(a) was an independent risk factor for cardiovascular and cerebrovascular events (adjusted HR = 1.68, 95% CI: 1.03–2.72, *p* = 0.037) and an independent risk factor for stroke (adjusted HR = 1.77, 95% CI: 1.06–2.96, *p* = 0.029). Elevated Lp(a) was associated with increased MACE risk only in patients with atherosclerotic thrombotic stroke (annual incidence 25.8% vs. 14.0%, log‐rank *p* = 0.041) (Hoshino et al. 2025). Arboix et al. (2010) found that ischemic lacunar stroke differs markedly from other acute ischemic cerebrovascular diseases: the former represents small vessel disease caused by occlusion of a single perforating artery, without significant cortical ischemia or severe carotid stenosis, while the latter predominantly involves large vessel disease or cardioembolic events. Patients with lacunar stroke exhibited higher hypertension prevalence (69.4% vs. 48.4%), relatively younger age, and more frequent presentation of specific lacunar syndromes such as pure motor hemiplegia. Regarding prognosis, lacunar stroke demonstrated superior short‐term outcomes with extremely low in‐hospital mortality. Our study similarly found a lower proportion of lacunar stroke patients (39.19%) among those with poorer prognosis (*p* < 0.05) (Arboix et al. 2010).

Furthermore, multiple studies indicate that a higher NIHSS score at admission is an independent risk factor for poor prognosis in acute cerebral infarction patients (Inoue et al. 2022; Takano et al. 2024; Y. Wang et al. 2024). Similarly, cardioembolic stroke (and atherosclerotic thrombotic stroke) represents the ischemic infarction subtype with the highest in‐hospital mortality rate (Pujadas Capmany et al. 2004). Compared to other ischemic stroke subtypes, patients with cardioembolic stroke exhibit poorer short‐term prognosis (Pujadas Capmany et al. 2004). Therefore, this study not only excluded patients with cardioembolic stroke to avoid potential confounding but also revealed through multivariate logistic regression analysis that LPA remained significantly associated with prognosis in acute cerebral infarction patients after adjusting for multiple confounding factors. This finding suggests the potential value of LPA in assessing prognosis for acute cerebral infarction, providing a reference basis for optimizing clinical risk stratification and individualized interventions.

Lp(a) influences cerebral infarction prognosis through numerous potential mechanisms. Previous studies have revealed that Lp(a) directly promotes atherosclerosis via its unique structure—resembling LDL particles and containing oxidized phospholipids from Apo(a). Its adhesion to the vascular wall activates endothelial cells, induces monocyte infiltration and transformation into macrophages, and accelerates foam cell formation. Furthermore, Lp(a) enhances thrombogenicity by competitively inhibiting plasminogen activity, potentially exacerbating thrombotic events associated with large‐artery atherosclerosis (LAA) during acute cerebral infarction. Similarly, oxidized phospholipids carried by Lp(a) induce endothelial cell apoptosis and promote the release of inflammatory mediators (e.g., IL‐6, TNF‐α) via the NF‐κB pathway (Arnold et al. 2021; Panagiotopoulos et al. 2025; Xu et al. 2022). Multiple studies indicate that combined detection of Lp(a) with inflammatory markers such as lipoprotein‐associated phospholipase A2 (Lp‐PLA2), myeloperoxidase (MPO), and high‐sensitivity C‐reactive protein (hs‐CRP) enhances predictive value for acute cerebral infarction prognosis (Hua et al. 2023; Youyou et al. 2023; Zhao et al. 2021).

This study possesses multiple advantages: First, it focuses on the association between Lp(a) and prognosis. Through multi‐level statistical methods—including univariate analysis, multinomial logistic regression (adjusting for confounding factors such as gender, age, and underlying diseases), threshold effect analysis, and ROC curve analysis—it systematically validated Lp(a)’s impact on adverse outcomes and its threshold values, comparing its predictive value with traditional indicators. Second, it incorporates comprehensive indicators spanning multidimensional variables, including lipid profiles, inflammation, vascular structure, neurological function scores, and underlying diseases, ensuring data processing reliability and scientific rigor. The study also has limitations: First, as a single‐center retrospective design with only 175 cases, selection bias may exist, requiring caution when extrapolating results to other regions or hospital populations. Second, dynamic changes in Lp(a) levels during treatment were not collected, precluding assessment of the impact of lipid‐lowering or antiplatelet therapies on Lp(a) levels and prognosis. In addition, variables such as smoking duration, alcohol consumption, and medication details (e.g., statin dosage) were not recorded, potentially overlooking potential confounding factors. Third, all enrolled patients survived during the follow‐up period with no in‐hospital deaths. Consequently, Lp(a) characteristics in deceased patients could not be analyzed, nor could the association between Lp(a) and mortality risk be explored. This limitation hinders a comprehensive assessment of Lp(a)’s value in predicting long‐term outcomes for AIS patients. In addition, existing data cannot assess the impact of treatment on Lp(a) levels or the association between dynamic Lp(a) changes and long‐term outcomes (e.g., 1‐year recurrence rates). Future prospective studies are needed to supplement these data and further refine the application framework of Lp(a) in the prognostic management of acute cerebral infarction. Future research directions should include multicenter, large‐sample prospective cohort studies enrolling patients from diverse geographic regions and healthcare facilities. By integrating ischemic stroke classification using the TOAST subtype framework, these studies should clarify the specific prognostic predictive value of Lp(a) for different subtypes (e.g., lacunar infarction, atherosclerotic thrombotic infarction), thereby enhancing the generalizability of findings. Second, explore the combined predictive value of Lp(a) with other biomarkers, such as inflammatory markers (e.g., C‐reactive protein), vascular injury markers (e.g., homocysteine), or imaging indicators (e.g., cerebral small vessel disease burden), to construct more precise prognostic assessment models and provide tools for clinical risk stratification. Third, conduct interventional studies to evaluate the impact of targeted therapies for reducing Lp(a) levels—such as novel lipid‐lowering drugs or lifestyle interventions—on short‐term functional recovery and long‐term recurrence/mortality risks in AIS patients, thereby establishing the clinical feasibility of Lp(a) as a therapeutic target. Fourth, delve into the underlying mechanisms by which Lp(a) influences stroke prognosis. Combine basic experiments with clinical research to explore the molecular pathways through which Lp(a) contributes to atherosclerosis, thrombosis, or neural injury, providing theoretical foundations for developing targeted intervention strategies. Fifth, focus on the distinct characteristics of specific populations, such as elderly patients (≥ 85 years old) or those with comorbidities like diabetes or chronic obstructive pulmonary disease (COPD). Analyze the differential associations between Lp(a) levels and outcomes in these subgroups to clarify the predictive utility of Lp(a) across different clinical subgroups. This will provide evidence for developing personalized prognostic assessment protocols and treatment strategies.

## 5 Conclusion

This study included 175 patients with acute cerebral infarction. After multivariate adjustment, Lp(a) remained an independent risk factor for poor prognosis (OR = 1.09, *p* = 0.004). The high Lp(a) group had a 3.89‐fold increased risk of adverse outcomes compared with the low‐level group (*p* = 0.013), with a significant dose–response trend. Threshold effect analysis showed that when Lp(a) exceeded 31.2 mg/dL, the risk of poor prognosis increased by 79%. The combined model of “Lp(a) plus admission NIHSS score” demonstrated better predictive performance (AUC = 0.6820, specificity = 0.9208) than either single indicator alone. Sensitivity analyses (including exclusion/imputation of missing data and *E‐*value testing) confirmed the robustness and reliability of the association. In clinical practice, Lp(a) can serve as an important biomarker for prognostic assessment of AIS, facilitating early risk stratification and individualized intervention.

## Author Contributions

**Xun He**: software. **Jie Hu**: software. **Yueyu Zhang**: conceptualization. **Xinyi Chen**: conceptualization. **Juncang Wu**: conceptualization, methodology. **Yi Tang**: validation, investigation. All authors approved the final version.

## Funding

This study was funded by the project of “Hefei 2022 Key Common Technology Research and Development Project” of the Science and Technology Innovation Policy (GJ2022SM07).

## Conflicts of Interest

The authors declare no conflicts of interest.

## Supporting information




**Supplementary Material**: brb371475‐sup‐0001‐SuppMat.docx

## Data Availability

Original data are available from the corresponding author on reasonable request.

## References

[brb371475-bib-0001] Arboix, A. , J. Massons , L. García‐Eroles , et al. 2010. “Nineteen‐Year Trends in Risk Factors, Clinical Characteristics and Prognosis in Lacunar Infarcts.” Neuroepidemiology 35, no. 3: 231–236. 10.1159/000319460. 20861654

[brb371475-bib-0002] Arnold, M. , J. Schweizer , C. T. Nakas , et al. 2021. “Lipoprotein(a) Is Associated With Large Artery Atherosclerosis Stroke Aetiology and Stroke Recurrence Among Patients Below the Age of 60 Years: Results From the BIOSIGNAL Study.” European Heart Journal 42, no. 22: 2186–2196. 10.1093/eurheartj/ehab081. 33709115

[brb371475-bib-0003] Arsenault, B. J. , and P. R. Kamstrup . 2022. “Lipoprotein(a) and Cardiovascular and Valvular Diseases: A Genetic Epidemiological Perspective.” Atherosclerosis 349: 7–16. 10.1016/j.atherosclerosis.2022.04.015. 35606078

[brb371475-bib-0004] Bhatia, H. S. , and M. J. Wilkinson . 2022. “Lipoprotein(a): Evidence for Role as a Causal Risk Factor in Cardiovascular Disease and Emerging Therapies.” Journal of Clinical Medicine 11, no. 20: 6040. 10.3390/jcm11206040. 36294361 PMC9604626

[brb371475-bib-0005] Gasull, T. , and A. Arboix . 2022. “Molecular Mechanisms and Pathophysiology of Acute Stroke: Emphasis on Biomarkers in the Different Stroke Subtypes.” International Journal of Molecular Sciences 23, no. 16: 9476. 10.3390/ijms23169476. 36012743 PMC9409332

[brb371475-bib-0006] Hong, X.‐C. , M.‐C. Shu , S.‐R. Bao , S.‐Y. Chen , Y.‐X. Weng , and S.‐L. Lin . 2025. “Analysis of Factors Influencing Poor Neurological Outcomes in Patients With Acute Ischemic Stroke.” Annals of Medicine 57, no. 1: 2458209. 10.1080/07853890.2025.2458209. 40538140 PMC12931334

[brb371475-bib-0007] Hoshino, T. , T. Mizuno , S. Arai , et al. 2025. “Residual Lipoprotein(a)‐Associated Risk in Patients With Stroke or Transient Ischemic Attack.” Atherosclerosis 405: 119231. 10.1016/j.atherosclerosis.2025.119231. 40339358

[brb371475-bib-0008] Hua, M. , W.‐Y. Chen , L.‐H. Wang , X.‐H. Zou , and L.‐L. Mao . 2023. “The Value of Serum Lp‐PLA2 Combined With MPO in the Diagnosis of Cerebral Infarction Caused by Large Artery Atherosclerosis.” Clinical Neurology and Neurosurgery 232: 107899. 10.1016/j.clineuro.2023.107899. 37467579

[brb371475-bib-0009] Inoue, M. , T. Ota , T. Hara , et al. 2022. “An Initial High National Institutes of Health Stroke Scale Score and Any Intracranial Hemorrhage Are Independent Factors for a Poor Outcome in Nonagenarians Treated With Thrombectomy for Acute Large Vessel Occlusion: The Tokyo/Tama‐REgistry of Acute Endovascular Thrombectomy (TREAT) Study.” World Neurosurgery 165: e325–e330. 10.1016/j.wneu.2022.06.038. 35717017

[brb371475-bib-0010] Jiang, X. , J. Xu , X. Hao , et al. 2021. “Elevated Lipoprotein(a) and Lipoprotein‐Associated Phospholipase A2 Are Associated With Unfavorable Functional Outcomes in Patients With Ischemic Stroke.” Journal of Neuroinflammation 18, no. 1: 307. 10.1186/s12974-021-02359-w. 34963487 PMC8715597

[brb371475-bib-0011] Kosmas, C. E. , M. D. Bousvarou , E. J. Papakonstantinou , E.‐A. Zoumi , and L. S. Rallidis . 2024. “Lipoprotein (a) and Cerebrovascular Disease.” Journal of International Medical Research 52, no. 7: 03000605241264182. 10.1177/03000605241264182. 39082245 PMC11295242

[brb371475-bib-0012] Nam, K.‐W. , J. H. Han , C. K. Kim , et al. 2024. “High Glycated Albumin Is Associated With Early Neurological Deterioration in Patients With Acute Ischemic Stroke.” BMC Neurology 24, no. 1: 278. 10.1186/s12883-024-03747-4. 39127620 PMC11316286

[brb371475-bib-0013] Panagiotopoulos, E. , L. Palaiodimou , A. Theodorou , et al. 2025. “Lipoprotein(a) as a Stroke Biomarker: Pathophysiological Pathways and Therapeutic Implications.” Journal of Clinical Medicine 14, no. 9: 2990. 10.3390/jcm14092990. 40364021 PMC12072530

[brb371475-bib-0014] Pavlatos, N. , and D. K. Kalra . 2024. “The Role of Lipoprotein(a) in Peripheral Artery Disease.” Biomedicines 12, no. 6: 1229. 10.3390/biomedicines12061229. 38927436 PMC11200468

[brb371475-bib-0015] Pujadas Capmany, R. , A. Arboix , R. Casañas‐Muñoz , and N. Anguera‐Ferrando . 2004. “Specific Cardiac Disorders in 402 Consecutive Patients With Ischaemic Cardioembolic Stroke.” International Journal of Cardiology 95, no. 2–3: 129–134. 10.1016/j.ijcard.2003.02.007. 15193810

[brb371475-bib-0016] Raitakari, O. , A. Kivelä , K. Pahkala , et al. 2022. “Long‐Term Tracking and Population Characteristics of Lipoprotein (a) in the Cardiovascular Risk in Young Finns Study.” Atherosclerosis 356: 18–27. 10.1016/j.atherosclerosis.2022.07.009. 35961208

[brb371475-bib-0017] Takano, Y. , M. Koyanagi , T. Takamatsu , et al. 2024. “Clinical Evaluation of Mechanical Thrombectomy for Patients With Posterior Circulation Occlusion: A Retrospective Study.” Clinical Neurology and Neurosurgery 237: 108133. 10.1016/j.clineuro.2024.108133. 38340428

[brb371475-bib-0018] Wang, J. , X. Zhang , J. Tian , H. Li , H. Tang , and C. Yang . 2022. “Predictive Values of Systemic Inflammatory Responses Index in Early Neurological Deterioration in Patients With Acute Ischemic Stroke.” Journal of Integrative Neuroscience 21, no. 3: 94. 10.31083/j.jin2103094. 35633175

[brb371475-bib-0019] Wang, R. , W. Kong , and W. Zhang . 2024. “Serum Lipoprotein(a) as Predictive Factor for Early Neurological Deterioration of Acute Ischemic Stroke After Thrombolysis.” International Journal of General Medicine 17: 3791–3798. 10.2147/IJGM.S475767. 39239148 PMC11375750

[brb371475-bib-0020] Wang, Y. , X. Yuan , Y. Kang , L. Yu , W. Chen , and G. Fan . 2024. “Clinical Predictors of Prognosis in Stroke Patients After Endovascular Therapy.” Scientific Reports 14, no. 1: 667. 10.1038/s41598-024-51356-5. 38182739 PMC10770320

[brb371475-bib-0021] Xu, J. , X. Hao , R. Zhan , et al. 2022. “Effect of Lipoprotein(a) on Stroke Recurrence Attenuates at Low LDL‐C (Low‐Density Lipoprotein) and Inflammation Levels.” Stroke 53, no. 8: 2504–2511. 10.1161/STROKEAHA.121.034924. 35410491

[brb371475-bib-0022] Youyou, Z. , J. Ruirui , W. Hui , and L. Zhaoyang . 2023. “Association Between Lipoprotein(a) and Ischemic Stroke: Fibrinogen as a Mediator.” Journal of the Neurological Sciences 452: 120738. 10.1016/j.jns.2023.120738. 37517272

[brb371475-bib-0023] Yuan, S. , F. Li , H. Zhang , et al. 2024. “Impact of High Lipoprotein(a) on Long‐Term Survival Following Coronary Artery Bypass Grafting.” Journal of the American Heart Association 13, no. 3: e031322. 10.1161/JAHA.123.031322. 38240214 PMC11056181

[brb371475-bib-0024] Zhao, X. , M. Zhao , B. Pang , Y. Zhu , and J. Liu . 2021. “Diagnostic Value of Combined Serological Markers in the Detection of Acute Cerebral Infarction.” Medicine 100, no. 36: e27146. 10.1097/MD.0000000000027146. 34516506 PMC8428755

