# Supplementary material for: Clinical Study on the Association Between Lipoprotein(a) Levels and Prognosis in Patients With Acute Ischemic Stroke
Source: Brain Behav. 2026 May 11;16(5):e71475. doi: 10.1002/brb3.71475 (PMC13158877; doi:10.1002/brb3.71475)
Supplement: Supplementary file 1 — Supplementary Material: brb371475‐sup‐0001‐SuppMat.docx [file BRB3-16-e71475-s001.docx]

TableS1 Best threshold analysis of lipoprotein(a) and outcome prognosis

| Test | Best threshold | Specificity | Sensitivity | Accuracy | Positive-LR | Negative-LR | Diagnose-OR | N-for-diagnose | Postive-pv | Negative-pv |
| --- | --- | --- | --- | --- | --- | --- | --- | --- | --- | --- |
| Both | 0.2759 | 0.9208 | 0.3649 | 0.6857 | 4.6064 | 0.6898 | 6.6782 | 3.5007 | 0.7714 | 0.6643 |
| LPA | 19.88 | 0.7129 | 0.6081 | 0.6686 | 2.1179 | 0.5497 | 3.8526 | 3.1155 | 0.6081 | 0.7129 |
| Admission NIHSS score | 3.5 | 0.6337 | 0.5676 | 0.6057 | 1.5493 | 0.6824 | 2.2703 | 4.9694 | 0.5316 | 0.6667 |

TableS2 The association between LPA and outcomes in different stroke types

| Variables | n (%) | OR (95%CI) | P | P for interaction |
| --- | --- | --- | --- | --- |
| All patients | 175 (100.00) | 1.09 (1.03, 1.15) | 0.0042 |  |
| Stroke Type |  |  |  | 0.901 |
| Non-lacunar ischemic stroke | 76 (43.43) | 1.16 (1.04 ~ 1.30) | 0.007 |  |
| Lacunar ischemic stroke | 99 (56.57) | 1.07 (0.93 ~ 1.23) | 0.368 |  |

Adjust for:gender; age; smoke; drink; hypertension; diabetes; heart disease; stroke; weight; height; activity; SBP;DBP;TOAST Classification

Table S3 Sensitivity analysis of logistic regression after including patients who received thrombolysis and thrombectomy

| Exposure | Model1 | Model2 | Model3 |
| --- | --- | --- | --- |
| Lpa continuous variable | OR (95%CI) Pvalue | OR (95%CI) Pvalue | OR (95%CI) Pvalue |
| Lpa grouping variable |  |  |  |
| Low group | Ref. | Ref. | Ref. |
| Medium group | 0.95 (0.43, 2.07) 0.8923 | 1.27 (0.43, 3.73) 0.6679 | 1.19 (0.40, 3.57) 0.7584 |
| High group | 3.21 (1.51, 6.86) 0.0025 | 3.39 (1.20, 9.60) 0.0217 | 3.44 (1.21, 9.73) 0.0202 |
| Trend | 0.0022 | 0.0175 | 0.0155 |

OR: odds ratio; CI: confidence interval; Ref: reference.

Model1 adjust for: None

Model2 adjust for:gender; age; smoke; drink; hypertension; diabetes; heart disease; stroke; weight; height; activity; SBP;DBP

Model3 adjust for:gender; age; smoke; drink; hypertension; diabetes; heart disease; stroke; weight; height; activity; SBP;DBP;TOAST Classification;IVT/EVT;

Table S4 Sensitivity analysis of logistic regression after including missing lipoprotein(a) data

| Exposure | Model1 | Model2 | Model3 |
| --- | --- | --- | --- |
| Lpa continuous variable | OR (95%CI) Pvalue | OR (95%CI) Pvalue | OR (95%CI) Pvalue |
| Lpa grouping variable |  |  |  |
| Low group | Ref. | Ref. | Ref. |
| Medium group | 1.06 (0.48, 2.31) 0.8923 | 1.72 (0.58, 5.04) 0.3261 | 1.64 (0.54, 4.98) 0.3804 |
| High group | 3.39 (1.58, 7.29) 0.0018 | 4.10 (1.39, 12.09) 0.0104 | 4.14 (1.40, 12.25) 0.0102 |
| Trend | 0.0015 | 0.0097 | 0.0090 |

OR: odds ratio; CI: confidence interval; Ref: reference.

Model1 adjust for: None

Model2 adjust for:gender; age; smoke; drink; hypertension; diabetes; heart disease; stroke; weight; height; activity; SBP;DBP

Model3 adjust for:gender; age; smoke; drink; hypertension; diabetes; heart disease; stroke; weight; height; activity; SBP;DBP;TOAST Classification

Table S5: Missing data for all participants included

| Patient exclusion flow diagram | Reason_for_exclusion | Number_excluded | Remaining | Percentage_of_original |
| --- | --- | --- | --- | --- |
| 1 | Patients receiving thrombolysis or thrombectomy | 12 | 186 | 6.1% |
| 2 | Lost to follow-up or death | 3 | 183 | 1.5% |
| 3 | Missing LPA data | 6 | 177 | 3.0% |
| 4 | Missing complication information | 2 | 175 | 1.0% |
|  | Total excluded | 23 | 175 | 11.6% |
